# Design, synthesis, and evaluation of novel 2-phenylpropionic acid derivatives as dual COX inhibitory-antibacterial agents

**DOI:** 10.1080/14756366.2017.1310726

**Published:** 2017-04-16

**Authors:** Hülya Karaca Gençer, Ulviye Acar Çevik, Betül Kaya Çavuşoğlu, Begüm Nurpelin Sağlık, Serkan Levent, Özlem Atlı, Sinem Ilgın, Yusuf Özkay, Zafer Asım Kaplancıklı

**Affiliations:** a Department of Pharmaceutical Microbiology, Faculty of Pharmacy, Anadolu University, Eskişehir, Turkey;; b Department of Pharmaceutical Chemistry, Faculty of Pharmacy, Anadolu University, Eskişehir, Turkey;; c Doping and Narcotic Compounds Analysis Laboratory, Faculty of Pharmacy, Anadolu University, Eskişehir, Turkey;; d Department of Pharmaceutical Toxicology, Faculty of Pharmacy, Anadolu University, Eskişehir, Turkey

**Keywords:** Phenylpropionic acid, COX inhibition, antibacterial, dual effect, docking

## Abstract

A series of 2-(4-substitutedmethylphenyl)propionic acid derivatives **(6a–6m)** were synthesized, characterized and evaluated for cyclooxygenase (COX) enzyme inhibitory and antimicrobial activity. Test compounds that exhibited good COX inhibition and antibacterial activity were further screened for their cytotoxicity and genotoxicity. Compounds **6h** and **6l** showed better COX-1 and COX-2 inhibition when compared to ibuprofen. Inhibition potency of these compounds against COX-2 was very close to that of nimesulide. The compounds **6d, 6h, 6l** and **6m** displayed promising antibacterial property when compared to chloramphenicol. However, the compound **6l** was emerged as the best dual COX inhibitory-antibacterial agent in this study. The ADME prediction of the compounds revealed that they may have a good pharmacokinetic profile. Docking results of the compounds **6h** and **6l** with COX-1 (PDB ID: 1EQG) also exhibited a strong binding profile.

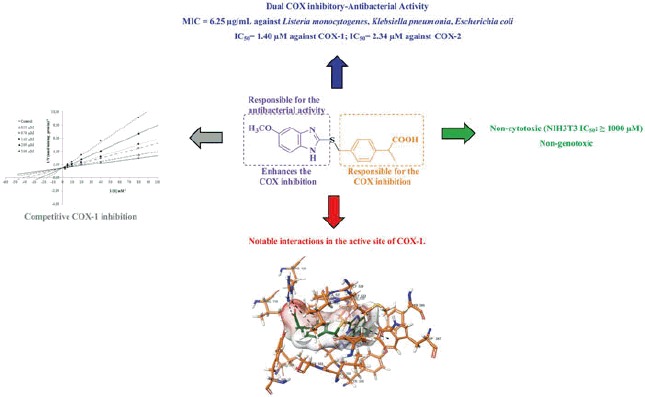

## 1. Introduction

Prostaglandin H synthase (PGHS) also known as cyclooxygenase (COX) is a dimeric membrane enzyme that is in charge of production of prostaglandins, prostacyclins and thromboxanes^1^. Prostaglandins are lipid autacoids associated with physiologic and pathologic processes, including inflammation^2^. Nonsteroidal anti-inflammatory drugs (NSAIDs) are the most prescribed medicines for the therapy of diverse inflammatory diseases. The mechanism of action of NSAIDs is based on the repression of prostaglandin biosynthesis from arachidonic acid via inhibiting the enzyme COXs^3^.

COX has both COX and peroxidase activities. The COX activity of COX enzymes forms PGG2 by incorporation of two oxygen molecules to arachidonic acid via catalytic residue Tyr385. As a consequence of peroxidase activity of COXs, PGG2 is reduced to PGH2 that is converted to prostaglandins, prostacyclins and thromboxanes^4^. Two isoforms^5^ of COX exist, COX-1 and COX-2. The constitutive COX-1 isoform is produced in most tissues and responsible for the synthesis of cytoprotective PGs in the gastrointestinal system, vascular homeostasis and platelet aggregation, whereas the inducible COX-2 is expressed in some tissues in order to produce prostaglandins thus initializes the inflammation^6^.

The structures and sequence of COX-1 and COX-2 enzymes are quite similar. Each enzyme consists of three structural domains: N-terminal epidermal growth factor (EGF) domain, membrane-binding motif and C-terminal catalytic domain that includes both the COX and peroxidase active sites^7^. Ibuprofen, flurbiprofen and naproxen are prominent members of NSAIDs containing 2-arylpropionic acid scaffold. Depending on numerous studies, it is regarded that the free carboxylic acid group situated in these molecules composes critical interactions with Arg120, Glu524 and Tyr355 in the COX active site^8–10^. The carboxylic acid structure, hence, is considered as an essential pharmacophoric core for COX activity^11^. According to studies, esterification or amidation of the free carboxylic acid group cause reduced COX inhibition activity^12^.

Azole compounds are electron-rich nitrogen heterocycles, playing an extremely essential role in medicinal area. Hence, they have been gained a special attention^13^. Due to their heteroatomic ring system and electron-rich property, azole-based compounds can easily interact with the enzymes and receptors in organisms as a result of coordination bonds, hydrogen bonds, ion-dipole, cation–π, π–π stacking and hydrophobic effect as well as van der Waals force, etc., thereby exhibiting various bioactivities^14^. The design, synthesis and antimicrobial activity of azole derivatives have been widely examined and have become one of the highly important highlights in recent years, and the progress is quite rapid. Particularly, a large number of azole-based antibacterial and antifungal compounds have been penetratingly studied as candidates and even some of them have been used at the clinic, which have indicated the excessive potential and development value of azole compounds^15–22^. Furthermore, azole-based compounds were reported to exhibit biologically important activities as anti-inflammatory and analgesic agents^23^.

Markedly, inflammation and infection are not identical, even in the case where infection is the primary reason of the inflammation. Moreover, the inflammatory response elicited by an invading organism can result in host damage, raise the availability of nutrients and facilitate access to host tissues. Additionally, inflammation may cause accumulation of fluid in the injured area, which may stimulate bacterial growth^24^. Other reports revealed that NSAIDs may increase the progression of bacterial infection^25^^,^^26^. Furthermore, in the management of infectious and inflammatory diseases, the use of multidrug therapy is an increasing concern for patients with damaged liver or kidney functions, patients with diseases of the gastrointestinal system or patients suffering from diverse side effects of other drugs. Monotherapy would be preferred with regards to both the pharmacoeconomics and the patient compliance^27^. Therefore, a dual COX inhibitory-antibacterial agent with an improved safety profile is necessary for enhanced therapeutic benefits and better patient compliance. Prompted from this requirement lot of studies have been reported^28–43^.

As a result, above-mentioned information directed us to synthesize some novel 2-(4-substitutedmethylphenyl)propionic acid derivatives and investigate their inhibitory activity against COX-1, COX-2 enzymes and various microbial strains.

## 2. Experimental

### 2.1. Materials and methods

Entire chemicals used in the syntheses were purchased from Sigma-Aldrich Chemicals (Sigma-Aldrich Corp., St. Louis, MO) or Merck Chemicals (Merck KGaA, Darmstadt, Germany). Melting points of the synthesized compounds were determined by MP90 digital melting point apparatus (Mettler Toledo, Columbus, OH) and were uncorrected. ^1^H NMR and ^13^C NMR spectra were recorded by a Bruker 300 and 75 MHz digital FT-NMR spectrometer (Bruker Bioscience, Billerica, MA) in DMSO-d_6_, respectively. In the NMR spectra, splitting patterns were designated as follows: s: singlet; d: doublet; t: triplet; and m: multiplet. Coupling constants (*J*) were reported as Hertz. The IR spectra were obtained on a Shimadzu, IR Affinity-1 S (Shimadzu, Tokyo, Japan). HRMS studies were performed on Shimadzu LCMS-IT-TOF system (Shimadzu, Tokyo, Japan). The purities of compounds were checked by TLC on silica gel 60 F254 (Merck KGaA, Darmstadt, Germany).

#### 2.1.1. Synthesis of 1-(4-methylphenyl) ethanol (1)

4-Methyl acetophenone (0.05 mol, 6.70 g) was dissolved in methanol (100 mL) and NaBH_4_ (0.05 mol, 1.89 g) was added in portions. Once the reaction was completed, methanol was evaporated and precipitated product was washed with water. The precipitate was extracted with dichloromethane in portions (3 × 100 mL), extracts were combined and dried with anhydrous sodium sulfate. The solvent was evaporated, and the residue was recrystallized from ethanol to give the 1-(4-methylphenyl) ethanol (**1**)^44^. Yield; 74%.

#### 2.1.2. Synthesis of 1-(4-methylphenyl)ethyl 4-methylbenzenesulfonate (2)

1-(4-Methylphenyl)ethanol (**1**) (0.04 mol, 5.45 g) and TEA (0.04 mol, 5.58 mL) in dichloromethane (100 mL) was taken in a saturated CaCl_2_ ice bath into ice bath. *p*-Tosyl chloride (0.012 mol, 2.29 g) in dichloromethane was added dropwise and the mixture was stirred for 40 h. The precipitated product was filtered, washed with 10% tartaric acid and then 5 N potassium chloride solution to give 1-(4-methylphenyl)ethyl 4-methylbenzenesulfonate (**2**). Yield; 72%^44^.

#### 2.1.3. Synthesis of 2-(4-methylphenyl) propionitrile (3)

1-(4-Methylphenyl)ethyl 4-methylbenzenesulfonate (**2**) (0.02 mol, 5.81 g) was dissolved in dimethyl sulfoxide (20 mL) and NaCN (0.02 mol, 0.98 g) was added. The solution was refluxed at 90 °C for 18 h. After completion of reaction, the mixture was poured into iced-water and extracted with diethyl ether in portions (3 × 100 mL). The extracts were combined and dried with anhydrous sodium sulfate. The solvent was evaporated to obtain 2-(4-methylphenyl) propionitrile (**3**). Yield; 68%^44^.

#### 2.1.4. Synthesis of 2-(4-methylphenyl) propionic acid (4)

2-(4-Methylphenyl)propionitrile (0.015 mol, 2.18 g) was dissolved in 5 N HCl (40 mL). The mixture was refluxed for 1 h. The precipitated product was extracted with ethyl acetate in portions (3 × 100 mL). The extracts were combined and dried with anhydrous sodium sulfate. The solvent was evaporated, and then raw product was recrystallized from ethanol to give 2-(4-methylphenyl) propionic acid (**4**)^44^. Yield; 78%.

#### 2.1.5. Synthesis of 2-(4-(bromomethyl)phenyl) propionic acid (5)

2-(4-Methylphenyl)propionic acid (0.01 mol, 1.64 g) was dissolved in ethyl acetate (50 mL) and catalytic amount of HBr was added. This solution was taken into ice bath and bromine (0.012 mol, 0.61 mL) in ethyl acetate (20 mL) was added dropwise. After completion of dropping the reaction mixture was stirred at room temperature for 2 h. The solvent was evaporated and precipitated product was washed with water, dried and then recrystallized from ethanol to afford 2-(4-(bromomethyl)phenyl) propionic acid (**5**). Yield; 79%^45^.

#### 2.1.6. General procedure for the synthesis of 2-(4-substitutedmethylphenyl)propionic acid (6a–6n)

2-(4-Bromo-methylphenyl) propionic acid (0.001 mol, 0.243 g) and appropriate (benz)azolylthiol derivative (0.001 mol) were dissolved in acetone. The solution was refluxed at 40 °C for 12 h. Acetone was evaporated, residue was washed with water, filtered, dried and recrystallized from ethanol to obtain final products (**6a–6n**)^46^.

##### 2.1.6.1. 2-(4-(((4,5-Dihydrothiazol-2-yl)thio)methyl)phenyl)propanoic acid (6a)

Yield: 77%, M.P.=154.2–156.3 °C, FTIR (ATR, cm^−1^): 3410 (O–H), 1701 (C=O), 1047, 845, 777. ^1^H-NMR (300 MHz, DMSO-d_6_): *δ* = 7.36 (2 H, d, *J* = 8.2 Hz, 1,4-disubs. benzene–CH–), 7.25 (2 H, d, *J* = 8.2 Hz, 1,4-disubs. benzene–CH–), 4.46 (2 H, s, –CH_2_–), 4.21 (1 H, t, *J* = 8.2 Hz, –CH_2_), 3.64 (1 H, q, *J* = 7.1 Hz, –CH–), 3.59 (1 H, t, *J* = 8.2 Hz, –CH_2_–), 1.33 (3 H, d, *J* = 7.1 Hz, –CH_3_). ^13^C-NMR (75 MHz, DMSO-d_6_): *δ* = 175.7, 156.1, 141.3, 134.9, 129.6, 128.2, 60.4, 44.8, 37.2, 35.1 and 18.9. HRMS (*m/z*): [M + H]^+^ calcd for C_13_H_15_NO_2_S_2_: 282.0617; found 282.0603.

##### 2.1.6.2. 2-(4-(((1-Methyl-1H-imidazol-2-yl)thio)methyl)phenyl)propanoic acid (6b)

Yield: 76%, M.P.=liquid, FTIR (ATR, cm^−1^): 3391 (O–H), 1717 (C=O), 1038, 860, 698. ^1^H-NMR (300 MHz, DMSO-d_6_): *δ* = 7.82 (1 H, d, *J* = 2.0 Hz, imidazole –CH–), 7.78 (1 H, d, *J* = 2.0 Hz, imidazole –CH–), 7.19 (2 H, d, *J* = 8.3 Hz, 1,4-disubs. benzene –CH–), 7.14 (2 H, d, *J* = 8.3 Hz, 1,4-disubs. benzene –CH–), 4.41 (2 H, s, –CH_2_–), 3.64 (1 H, q, *J* = 7.1 Hz, –CH–), 3.54 (3 H, s, –CH_3_), 1.34 (3 H, d, *J* = 7.1 Hz, –CH_3_). ^13^C-NMR (75 MHz, DMSO-d_6_): *δ* = 175.6, 141.6, 139.2, 135.1, 129.3, 128.2, 125.9, 121.6, 44.8, 35.3 and 18.9. HRMS (*m/z*): [M + H]^+^ calcd for C_14_H_16_N_2_O_2_S: 277.1005; found 277.1000.

##### 2.1.6.3. 2-(4-(((1H-1,2,4-Triazol-3-yl)thio)methyl)phenyl)propanoic acid (6c)

Yield: 85%, M.P.=liquid, FTIR (ATR, cm^−1^): 3393 (O–H), 1717 (C=O), 1022, 858. ^1^H-NMR (300 MHz, DMSO-d_6_): *δ* = 9.02 (1 H, d, *J* = 8.2 Hz, triazole –CH–), 7.18 (2 H, d, *J* = 8.2 Hz, 1,4-disubs. benzene –CH–), 7.15 (2 H, d*, J* = 8.3 Hz, 1,4-disubs. benzene –CH–), 4.41 (2 H, s, –CH_2_), 3.64 (1 H, q, *J* = 7.1 Hz, –CH–), 1.29 (3 H, d, *J* = 7.1 Hz, –CH_3_). ^13^C-NMR (75 MHz, DMSO-d_6_): *δ* = 175.7, 153.6, 141.2, 135.4, 129.5, 127.7, 44.8, 35.5 and 19.0. HRMS (*m/z*): [M + H]^+^ calcd for C_12_H_13_N_3_O_2_S: 264.0801; found 264.0789.

##### 2.1.6.4. 2-(4-(((4-Methyl-4 H-1,2,4-triazol-3-yl)thio)methyl)phenyl)propanoic acid (6d)

Yield: 81%, M.P.=liquid, FTIR (ATR, cm^−1^): 3420 (O–H), 1721 (C=O), 1024, 822, 760. ^1^H-NMR (300 MHz, DMSO-d_6_): *δ* = 8.28 (1 H, s, triazole –CH–), 7.19 (2 H, d, *J* = 8.3 Hz, 1,4-disubs. benzene –CH–), 7.14 (2 H, d, *J* = 8.3 Hz, 1,4-disubs. benzene –CH–), 4.41 (2 H, s, –CH_2_–), 3.64 (1 H, q, *J* = 7.1 Hz, –CH–), 3.55 (3 H, s, –CH_3_), 1.29 (3 H, d, *J* = 7.1 Hz, –CH_3_). ^13^C-NMR (75 MHz, DMSO-d_6_): *δ* = 175.6, 156.8, 143.2, 140.7, 136.8, 130.0, 127.6, 44.7, 35.3, 27.03 and 18.9. HRMS (*m/z*): [M + H]^+^ calcd for C_13_H_15_N_3_O_2_S: 278.0958; found 278.0952.

##### 2.1.6.5. 2-(4-(((1-Methyl-1 H-tetrazol-5-yl)thio)methyl)phenyl)propanoic acid (6e)

Yield: 82%, M.P.=liquid, FTIR (ATR, cm^−1^): 3374 (O–H), 1717 (C=O), 1038, 860, 698. ^1^H-NMR (300 MHz, DMSO-d_6_): *δ* = 7.31 (2H, d, *J* = 8.2 Hz, 1,4-disubs. benzene –CH–), 7.19 (2 H, d, *J* = 8.1 Hz, 1,4-disubs. benzene –CH–), 4.45 (2 H, s, –CH_2_–), 3.77 (3 H, s, –CH_3_), 3.61 (1 H, q, *J* = 7.1 Hz, –CH–), 1.28 (3 H, d*, J* = 7.1 Hz, –CH_3_. ^13^C-NMR (75 MHz, DMSO-d_6_): *δ* = 175.7, 153.6, 141.2, 135.4, 129.5, 128.0, 44.8, 34.0 and 18.8. HRMS (*m/z*): [M + H]^+^ calcd for C_12_H_14_N_4_O_2_S: 279.0910; found 279.0912.

##### 2.1.6.6. 2-(4-((Benzo[d]thiazol-2-ylthio)methyl)phenyl)propanoic acid (6f)

Yield: 80%, M.P.=128.2–130.8 °C. FTIR (ATR, cm^−1^): 3120 (O–H), 1734 (C=O), 1067, 854, 742. ^1^H-NMR (300 MHz, DMSO-d_6_): *δ* = 13.87 (1 H, s, –COOH), 7.62–7.67 (2 H, m, BT –CH–), 7.45 (2 H, d, *J* = 8.1 Hz, 1,4-disubs. benzene –CH–), 7.34–7.32 (2 H, m, BT –CH–), 7.25 (2 H, d, *J* = 8.1 Hz, 1,4-disubs. benzene –CH–), 4.67 (2 H, s, –CH_2_–), 3.67 (1 H, q, *J* = 7.4 Hz, –CH–),1.34 (3 H, d, *J* = 7.4 Hz, –CH_3_). ^13^C-NMR (75 MHz, DMSO-d_6_): *δ* = 175.7, 164.3, 151.7, 141.2, 135.5, 131.2, 129.9, 129.6, 128.1, 125.1, 118.8, 110.7, 44.8, 35.7 and 18.9. HRMS (*m/z*): [M + H]^+^ calcd for C_17_H_15_NO_3_S: 330.0617; found 330.0617.

##### 2.1.6.7. 2-(4-(((5-Chlorobenzo[d]thiazol-2-yl)thio)methyl)phenyl)propanoic acid (6g)

Yield: 85%, M.P.=162.3–164.7 °C, FTIR (ATR, cm^−1^): 3030 (O–H), 1715 (C=O), 1063, 860, 799. ^1^H-NMR (500 MHz, DMSO-d_6_): *δ* = 8.06 (1 H, d, *J* = 8.6 Hz, benzothiazole –CH–), 7.98 (1 H, d*, J* = 2.0 Hz, benzothiazole –CH–), 7.47 (2 H, d, *J* = 8.2 Hz, 1,4-disubs. benzene –CH–), 7.43 (1 H, dd, *J* = 8.6–2.0 Hz, benzothiazole –CH–), 7.27 (2 H, d, *J* = 8.2 Hz, 1,4-disubs. benzene –CH–), 4.64 (2 H, s, –CH_2_–), 3.67 (1 H, q, *J* = 7.2 Hz, –CH–), 1.35 (3 H, d, *J* = 7.2 Hz, –CH_3_). ^13^C-NMR (125 MHz, DMSO-d_6_): *δ* = 175.7, 169.5, 154.0, 141.2, 135.3, 133.9, 131.7, 129.7, 128.2, 125.0, 123.7, 121.1, 44.8, 36.8 and 18.9. HRMS (*m/z*): [M + H]^+^ calcd for C_17_H_14_NO_2_S_2_Cl: 364.0227; found 364.0218.

##### 2.1.6.8. 2-(4-(((5-Methoxybenzo[d]thiazol-2-yl)thio)methyl)phenyl)propanoic acid (6 h)

Yield: 77%, M.P.=166.5–168.2 °C, FTIR (ATR, cm^−1^): 3071 (O–H), 1724 (C=O), 1082, 835, 694. ^1^H-NMR (500 MHz, DMSO-d_6_): *δ* = 7.87 (1 H, d, *J* = 8.8 Hz, benzothiazole –CH–), 7.46–7.45 (3 H, m, 1,4-disubs. benzene –CH–, benzothiazole –CH–), 7.27 (2 H, d, *J* = 8.1 Hz, 1,4-disubs. benzene –CH–), 7.01 (1 H, dd, *J* = 8.0–2.5 Hz, benzothiazole –CH–), 4.62 (2 H, s, –CH_2_–), 3.84 (3 H, s, –OCH_3_), 3.67 (1 H, q, *J* = 7.1 Hz, –CH–), 1.35 (3 H, d*, J* = 7.1 Hz, –CH_3_).^13^C-NMR (125 MHz, DMSO-d_6_): *δ* = 175.6, 158.0, 148.5, 141.6, 134.6, 134.5, 129.5, 128.4, 128.0, 115.1, 114.7, 96.6, 56.4, 44.8, 37.0 and 18.9. HRMS (*m/z*): [M + H]^+^ calcd for C_18_H_17_NO_3_S_2_: 360.0723; found 360.0724.

##### 2.1.6.9. 2-(4-(((1H-benzo[d]imidazol-2-yl)thio)methyl)phenyl)propanoic acid (6i)

Yield: 78%, M.P.=208.7–210.9 °C, FTIR (ATR, cm^−1^): 3142 (O–H), 1699 (C=O), 1072, 851, 735. ^1^H-NMR (500 MHz, DMSO-d_6_): *δ* = 7.69–7.66 (2 H, m, benzimidazole –CH–), 7.44–7.41 (4 H, m, 1,4-disubs. benzene –CH–, benzimidazole –CH–), 7.27 (2 H, d, *J* = 8.0 Hz, 1,4-disubs. benzene –CH–), 4.72 (2 H, s, –CH_2_–), 3.66 (1 H, q, *J* = 7.1 Hz, –CH–). ^13^C-NMR (125 MHz, DMSO-d_6_): *δ* = 175.6, 150.2, 141.6, 134.7, 134.6, 129.5, 128.4, 125.0, 113.9, 44.8, 35.2 and 18.9. HRMS (*m/z*): [M + H]^+^ calcd for C_17_H_16_N_2_O_2_S: 313.1005; found 313.1009.

##### 2.1.6.10. 2-(4-(((5-Methyl-1H-benzo[d]imidazol-2-yl)thio)methyl)phenyl)propanoic acid (6j)

Yield: 80%, M.P.=209.1–211.6 °C, FTIR (ATR, cm^−1^): 3051 (O–H), 1701 (C=O), 1070, 856, 799. ^1^H-NMR (500 MHz, DMSO-d_6_): *δ* = 7.58 (1 H, d, *J* = 8.4 Hz, benzimidazole –CH–), 7.49 (1 H, s, benzimidazole –CH–), 7.40 (2 H, d*, J* = 7.9 Hz, 1,4-disubs. benzene –CH–), 7.30–7.26 (3 H, m, 1,4-disubs. benzene –CH–, benzimidazole –CH–), 4.73 (2 H, s, –CH_2_–), 3.66 (1 H, q, *J* = 7.1 Hz, –CH–), 2.47 (3 H, s, –CH_3_), 1.33 (3 H, d, *J* = 7.1 Hz, –CH_3_). ^13^C-NMR (125 MHz, DMSO-d_6_): *δ* = 175.6, 149.3, 141.6, 135.4, 134.5, 133.9, 129.5, 128.4, 126.8, 113.5, 113.3, 44.8, 36.8, 21.6 and 18.9. HRMS (*m/z*): [M + H]^+^ calcd for C_18_H_18_N_2_O_2_S: 327.1162; found 327.1149.

##### 2.1.6.11. 2-(4-(((5-Chloro-1H-benzo[d]imidazol-2-yl)thio)methyl)phenyl)propanoic acid (6k)

Yield: 81%, M.P.=208.8–211.5 °C, FTIR (ATR, cm^−1^): 3096 (O–H), 1703 (C=O), 1063, 851, 806. ^1^H-NMR (500 MHz, DMSO-d_6_): *δ* = 7.69 (1 H, s, benzimidazole –CH–), 7.61 (1 H, d*, J* = 8.6 Hz, benzimidazole –CH–), 7.41 (2 H, d, *J* = 7.9 Hz, 1,4-disubs. benzene –CH–), 7.35 (1 H, d, *J* = 8.6 Hz, benzimidazole –CH–), 7.26 (2 H, d, *J* = 7.9 Hz, 1,4-disubs. benzene –CH–), 4.68 (2 H, s, –CH_2_–), 3.66 (1 H, q, *J* = 7.1 Hz, –CH–), 1.33 (3 H, d*, J* = 7.1 Hz, –CH_3_). ^13^C-NMR (125 MHz, DMSO-d_6_): *δ* = 175.6, 152.0, 141.4, 137.4, 135.1, 129.5, 128.4, 128.2, 127.5, 124.1, 115.2, 113.9, 44.8, 36.1, 21.6 and 18.9. HRMS (*m/z*): [M + H]^+^ calcd for C_17_H_15_N_2_O_2_SCl: 347.0616; found 347.0608.

##### 2.1.6.12. 2-(4-(((5-Methoxy-1H-benzo[d]imidazol-2-yl)thio)methyl)phenyl)propanoic acid (6 l)

Yield: 82%, M.P.=175.6–178.4 °C, FTIR (ATR, cm^−1^): 3034 (O–H), 1730 (C=O), 1068, 845, 760. ^1^H-NMR (500 MHz, DMSO-d_6_): *δ* = 7.60 (1 H, d, *J* = 9.0 Hz, benzimidazole –CH–), 7.39 (2 H, d*, J* = 8.1 Hz, 1,4-disubs. benzene –CH–), 7.26 (2 H, d, *J* = 8.1 Hz, 1,4-disubs. benzene –CH–), 7.15 (1 H, d*, J* = 2.3 Hz, benzimidazole –CH–), 7.08 (1 H, dd, *J* = 9.0–2.3 Hz, benzimidazole –CH–), 4.72 (2 H, s, –CH_2_–), 3.85 (3 H, s, –OCH_3_), 3.66 (1 H, q, *J* = 7.2 Hz, –CH–), 1.33 (3 H, d, *J* = 7.2 Hz, –CH_3_). ^13^C-NMR (125 MHz, DMSO-d_6_): *δ* = 175.6, 158.0, 148.5, 141.6, 134.6, 134.5, 129.5, 128.4, 128.0, 115.1, 114.7, 96.6, 56.4, 44.8, 37.0 and 18.9. HRMS (*m/z*): [M + H]^+^ calcd for C_18_H_18_N_2_O_3_S: 343.1111; found 343.1099.

##### 2.1.6.13. 2-(4-(((5-Nitro-1H-benzo[d]imidazol-2-yl)thio)methyl)phenyl)propanoic acid (6 m)

Yield: 79%, M.P.=176.5–178.7 °C, FTIR (ATR, cm^−1^): 3098 (O–H), 1699 (C=O), 1063, 823, 748. ^1^^ ^H-NMR (500 MHz, DMSO-d_6_): *δ* = 8.35 (1 H, d*, J* = 1.0 Hz, benzimidazole –CH–), 8.08 (1 H, dd*, J* = 7.9–1.0 Hz, benzimidazole –CH–), 7.64 (1 H, d, *J* = 7.9 Hz, benzimidazole –CH–), 7.44 (2 H, d, *J* = 7.9 Hz, 1,4-disubs. benzene –CH–),7.25 (2 H, d, *J* = 7.8 Hz, 1,4-disubs. benzene –CH–), 4.63 (2 H, s, –CH_2_–), 3.65 (1 H, q, *J* = 7.1 Hz, –CH–), 1.34 (3 H, d, *J* = 7.1 Hz, –CH_3_). ^13^C-NMR (125 MHz, DMSO-d_6_): *δ* = 175.7, 156.4, 142.8, 141.0, 135.9, 129.5, 128.3, 128.1, 118.1, 113.9, 110.8, 44.8, 35.2 and 18.9. HRMS (*m/z*): [M + H]^+^ calcd for C_17_H_15_N_3_O_4_S: 358.0856; found 358.0858.

### 2.2. COX-1 and COX-2 inhibition assay

Inhibitory potency of the compounds against COX-1 and COX-2 enzymes was determined using fluorometric COX-1 and COX-2 inhibitor screening kits (Biovision, Zurich, Switzerland). Experimental protocol was followed as described in the guides of the supplier^47^^,^^48^. All of the pipettings in the assay were performed by Biotek Precision robotic system (BioTek Instruments, Inc., Winooski, VT). Fluorescence (Ex/Em =535/587 nm) of the samples were kinetically measured by BioTek-Synergy H1 multimode microplate reader (BioTek Instruments, Inc., Winooski, VT) at 25 °C for 5–10 min. Appropriate two points (T1 and T2) in the linear range of the plot were chosen, and the corresponding fluorescence values (RFU1 and RFU2) were obtained. The slope for all samples, including enzyme control (EC), by dividing the net ΔRFU (RFU2–RFU1) values by the time ΔT (T2–T1) were calculated by using following equation:

% Relative inhibition=(Slope of EC-Slope of S/Slope of EC)×100

This initial *in vitro* assay was done with two concentrations (10^−3^ and 10^−4^ M) for all compounds. The compounds, showing inhibition above 50%, were further assayed by the same protocol at varying concentrations (10^−5^ and 10^−9^ M) to determine their IC_50_ against COX-1 and COX-2 enzymes. The IC_50_ value was calculated from the plots of enzyme activity against concentrations by applying regression analyses on GraphPad Prism Version 5 (GraphPad Software, La Jolla, CA).

### 2.3. Enzyme kinetics

Enzyme kinetics study was performed to assess the nature of inhibition by the most active derivatives (**6h** and **6l**) on the COX-1 enzyme. The enzyme kinetics were determined, wherein the arachidonic acid substrate either in the absence or presence of selected derivatives at different concentrations (IC_50_/4, IC_50_/2, IC_50_, 2 × IC_50_ and 4 × IC_50_). The mode of inhibition was determined by following the Lineweaver–Burk double reciprocal plot analysis of the data and calculated as per the Michaelis–Menten kinetics. To understand the possible mode of action, *K*_m_ and *V*_max_ were also calculated. The slopes of the Lineweaver–Burk plots were plotted versus the inhibitor concentration, and the *K*_i_ values were determined from the x-axis intercept as inhibition constant − *K*_i_.

### 2.4. Antimicrobial activity

Microbiological studies were performed according to following guides: CLSI reference M07-A9 broth microdilution method^49^ for bacterial strains and EUCAST definitive (EDef 7.1) method^50^ for *Candida* species. Synthesized compounds were tested for their *in vitro* growth inhibitory activity against *Staphylococcus aureus* (ATCC 25923), *Enterococcus faecalis* (ATCC 29212), *Listeria monocytogenes* (ATCC 1911), *Klebsiella pneumoniae* (NCTC 9633), *Escherichia coli* (ATCC 35218), *E. coli* (ATCC 25922) *Candida albicans* (ATCC 24433) *Candida krusei* (ATCC 6258) and *Candida parapsilosis* (ATCC 22019). Chloramphenicol and ketoconazole were used as control drugs.

The cultures were obtained from the Mueller–Hinton broth (Difco) for the bacterial strains after overnight incubation at 37 °C. The yeasts were maintained in Roswell Park Memorial Institute (RPMI) after overnight incubation at 37 °C. The inocula of test microorganisms adjusted to match the turbidity of a Mac Farland 0.5 standard tube as determined with a spectrophotometer and the final inoculum size was 0.5–2.5 × 105 cfu/mL for antibacterial and antifungal assays. Testing was carried out in Mueller–Hinton broth and RPMI at pH =7, and the two-fold serial dilutions technique was applied. The last well on the microplates containing only inoculated broth was kept as controls and the last well with no growth of microorganism was recorded to represent the minimum inhibitory concentration (MIC) expressed in µg/mL. For both the antibacterial and antifungal assays, the compounds were dissolved in DMSO. Further dilutions of the compounds and standard drugs in test medium were prepared at the required quantities of 1000, 500, 250, 125, 62.5, 31.25, 15.6, 7.8, 3.9 and 1.95 µg/mL concentrations with Mueller–Hinton broth and RPMI mediums. The completed plates were incubated for 24 h. At the end of the incubation, resazurin (20 µg/mL) was added into each well and plates were incubated for 2 h. MIC values were determined using a microplate reader at 590 nm excitation, 560 nm emission.

### 2.5. Cytotoxicity test

Cytotoxicity was tested using the NIH/3T3 mouse embryonic fibroblast cell line (ATCC^®^ CRL-1658™, London, UK). NIH/3T3 cells were incubated according to the supplier’s recommendations. NIH/3T3 cells were seeded at 1 × 10^4^ cells into each well of 96-well plates. MTT assay was performed as previously described^51^^,^^52^. The compounds were tested between 1000 and 0.316 µM concentrations. Inhibition percentage was calculated for each concentration according to the formula below, and IC_50_ values were determined by plotting a dose-response curve of inhibition percentage versus compound concentrations tested^53^.

$$\%\;inhibition=100-\left(\frac{mean\;sample\times 100}{mean\;solvent}\right)$$

### 2.6. Genotoxicity test

The genotoxicity of the most effective compounds was determined by Ames assay using Ames MPF 98/100 mutagenicity assay sample kit (Xenometrix AG, Allschwil, Switzerland) as previously described^52^^,^^54^. *Salmonella typhimurium* strains, TA98 (frameshift mutations) and TA100 (base-pair substitutions), are used in this assay. Concentration range of the compounds was between 16 and 5000 µg/mL according to the previous guidelines^55^. Compounds were prepared in six different concentrations (5, 2.5, 1.25, 0.625, 0.3125 and 0.156 mg/mL) in DMSO. Mutagenic potential was determined in absence and presence of Aroclor™-1254 induced male Sprague–Dawley rat liver microsomal enzyme (S9) mix (Xenometrix AG, Switzerland). Positive controls without S9 mix were 2-Nitrofluorene (2 µg/mL) and 4-nitroquinoline *N*-oxide (0.1 µg/mL) whereas 1 and 2.5 µg/mL of 2-aminoanthracene solutions were used as positive controls with S9 against TA 98 and TA100, respectively. Solvent control was prepared with 4% DMSO. At the end of the experiment, revertant bacteria dropped the pH of solution and indicator medium color was changed to yellow. Yellow wells were counted as positive and compared with the negative control. Fold induction over the negative control and fold induction over the baseline were calculated. Fold induction over the negative control is the ratio of the mean number of positive wells for the dose concentration divided by the mean number of positive wells for the zero dose (negative) control. Fold induction over the baseline is the ratio of the mean number of positive wells for the dose concentration divided by zero dose baseline. The zero dose baseline is obtained by adding one standard deviation to the mean number of positive wells of the zero dose control. Mutagenicity was determined according to the criteria from previous studies^52^^,^^56^. For a value ≤3 and significant increases between two and three-fold the baselines were classified as a weak mutagen, and increases ≥ three-fold the baselines were classified as a mutagen. For a value >3 and significant increases between 1.5 and 2.5-fold the baselines were classified as a weak mutagen, and increases ≥2.5-fold the baselines were classified as a mutagen. As a rule, at least two adjacent doses with significant increases or a significant increase at the highest dose level should be observed for a mutagenic compound. All doses were compared according to Student’s t-test at *p* < .05 for statistical significance. Compounds, which did not have any of the properties mentioned above were classified as a non-mutagenic compound.

### 2.7. Theoretical calculation of ADME parameters

Some physicochemical parameters, which were used to evaluate ADME properties of the compounds (**6a–6m**) were analyzed by online Molinspiration property calculation program^57^.

### 2.8. Molecular docking

A structure based *in silico* procedure was applied to discover the binding modes of the compounds **6h** and **6l** to COX-1 enzyme active site. The crystal structures of COX-1 enzyme (PDB ID: 1EQG), crystallized with the reversible inhibitor ibuprofen, was retrieved from the Protein Data Bank server (www.pdb.org).

The docking study was performed by using *Maestro 10.6* software (Koingo Software, Inc., Kelowna, Canada)^58^. The X-ray crystal structure was submitted to the *Protein Preparation Wizard* protocol of the *Schrödinger Suite 2016 Update 3*^59^ to follow similar procedures described previously^60^. Ligand preparation was applied by the *LigPrep 3.8*^61^ to assign the protonation states and atom types of a molecule, correctly. The grid generation was formed using *Glide 7.1*^62^ program and docking runs were performed with single precision docking mode (SP).

## 3. Results and discussion

### 3.1. Chemistry

**Scheme 1.**
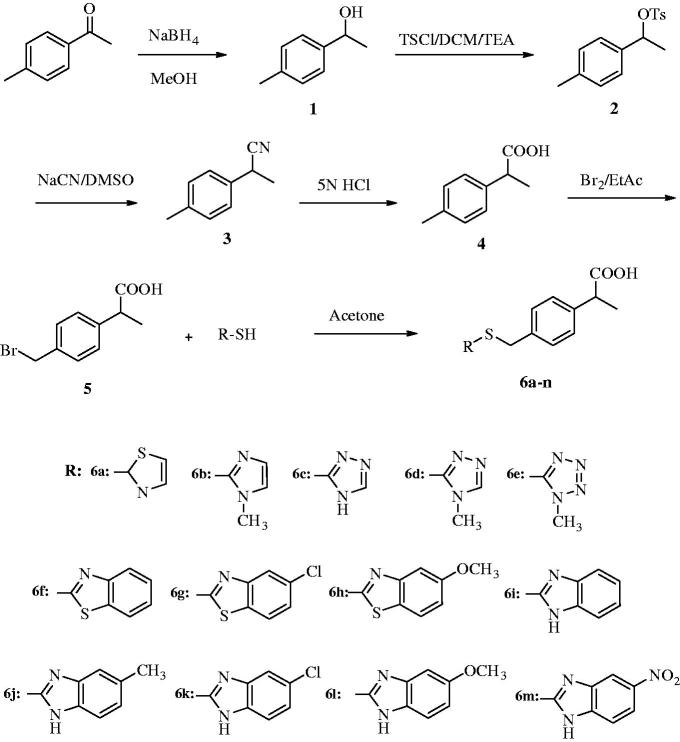
Synthesis way for target compounds (**6a–6m**).

Target compounds were synthesized in six steps following literature methods (Scheme 1). In the first step, 4-methylacetophenone (**1**) was reduced to 1-(4-methylphenyl)ethanol (**2**) in MeOH using NaBH_4_. Second, in a saturated CaCl_2_ ice bath, the compound **2** was treated with p-tosyl chloride to obtain 1-(4-methylphenyl)ethyl 4-methylbenzenesulfonate (**3**), which was reacted with NaCN to give 2-(4-methylphenyl)propionitrile **(4**) in the third step. Hydrolysis of compound **3** with 5 N HCl afforded the 2-(4-methylphenyl)propionic acid (**4**) in the next step. Bromination of the compound **4** in EtOAc gave the 2-(4-bromomethylphenyl)propionic acid (**5**), which was subjected to substitution reaction with various (benz)azolylthiols to obtain final compounds **6a**–**6m**. As a result of synthesis path, the intermediate compounds were obtained in varying yields of 68–79%, whereas final compounds were isolated in 76–85% yields. Structural elucidation of the synthesized compounds was performed by spectral analyses. In the IR spectra, O–H and C=O stretching absorption belonging to carboxylic acid group were observed over 3000 cm^−1^ as broad bands and around 1700 cm^−1^ as sharp bands, respectively. In the NMR spectra, –CH_3_ protons recorded as doublet at 1.28–1.35 ppm and –CH_3_ carbon was recorded at 18.8–19.0 ppm. A quartet peak at 3.61–3.67 ppm was observed for –CH– proton and carbon of –CH– was assigned at 34.0–37.0 ppm. Protons of –SCH_2_– were observed as singlet at 4.41–4.73 ppm and carbon of this group was recorded at 44.7–44.8 ppm. The O–H proton of carboxylic acid group was recorded as a singlet at 13.87 ppm in only compound **6f**, whereas the other compounds did not gave the same peak due to exchangeable carboxylic acid proton. Carbonyl carbon gave a peak at 175.6–175.7 ppm. All the other protons and carbons were recorded at expected values. All measured mass and isotope ratios were compatible with theoretical values in HRMS spectra.

### 3.2. COX enzymes inhibitory activity of the compounds

The *in vitro* COX-1 and COX-2 inhibitory activity of the compounds **6a**–**6m** was evaluated with a fluorescence-based COX assay (“COX-1 Fluorescent Inhibitor Screening Kit, Catalog No: K547-100” and “COX-2 Fluorescent Inhibitor Screening Kit, Catalog No: K548–100”, Biovision, Milpitas, CA) that utilizes the COX-mediated reduction of PGG_2_ to PGH_2_ to oxidize 10-acetyl-3,7-dihydroxyphenoxazine to resorufin. This highly fluorescent compound can easily be analyzed with an excitation wavelength of 530–540 nm and emission wavelength of 585–595 nm. The results of the COX inhibitory activity of the 2-(4-Substituted-methylphenyl)propionic acid derivatives (**6a–6m**) are summarized in the Table 1. Ibuprofen and nimesulide were used as nonselective COX inhibitor and selective COX-2 inhibitor, respectively. Selectivity indexes (SI) were expressed as IC_50_ (COX-1)/IC_50_ (COX-2). Selectivity toward COX-2 decreases as the corresponding SI decreases while selectivity toward COX-1 isoform increases as the corresponding SI decreases. It was noted that the compounds indicated SI of 0.52–0.63. This result suggested that the compounds had selectivity toward COX-1 isoenzyme. The compounds **6a–6e** indicated lower inhibition potency than the compounds **6f**–**6m** against both isoenzymes. It has been determined that compounds **6f, 6g, 6h** and **6l** have important inhibitory activity against both COX-1 and COX-2 enzymes. IC_50_ values of these compounds were comparable with that of nimesulide against the COX-2 enzyme. Furthermore, they were more effective than ibuprofen and nimesulide against COX-1 enzyme. The most active compounds **6h** and **6l** displayed IC_50_ values of 1.76 and 1.40 µM against COX-1 and IC_50_ values of 2.96 and 2.34 µM against COX-2.

**Table 1.** IC_50_ (μM) values of the compounds **4**, **6a–6m** and reference drugs against COX-1 and COX-2 enzymes.

Compound	IC_50_ (μM) COX-1	IC_50_ (μM) COX-2	^a^SI	Selectivity
**4**	38.23	64.30	0.59	COX-1
**6a**	22.16	37.79	0.59	COX-1
**6b**	28.61	47.42	0.60	COX-1
**6c**	19.47	36.68	0.53	COX-1
**6d**	24.65	39.95	0.62	COX-1
**6e**	21.77	36.29	0.60	COX-1
**6f**	2.36	4.41	0.54	COX-1
**6g**	2.46	4.25	0.58	COX-1
**6h**	1.76	2.96	0.59	COX-1
**6i**	5.90	9.56	0.62	COX-1
**6j**	4.40	7.53	0.58	COX-1
**6k**	3.71	5.91	0.63	COX-1
**6l**	1.40	2.34	0.60	COX-1
**6m**	4.48	8.66	0.52	COX-1
Ibuprofen	2.98	3.15	0.95	Nonselective
Nimesulide	4.28	1.35	3.17	COX-2

a The selectivity index (SI) was calculated as IC_50_ (COX-1)/IC_50_ (COX-2).

In order to observe contribution of variable groups to activity, COX inhibition potency of the intermediate product 2-(4-methylphenyl)propionic acid (**4**) was also evaluated. As seen in Table 1, the compound **4** has a lower potency than those of final compounds (**6a–6n**).

### 3.3. Enzyme kinetics

**Figure 1.**
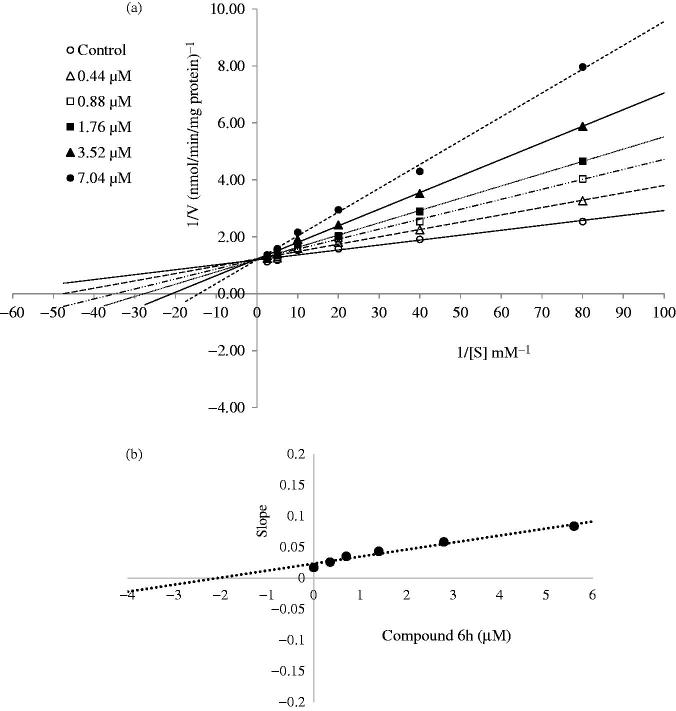
(a) Lineweaver–Burk plots for the inhibition of COX-1 enzyme by compound **6h**. [S], substrate concentration (mM); V, reaction velocity (nmol/min/mg protein). Inhibitor concentrations are shown at the left. *K*_m_ values from 4 × IC_50_ to Control_;_ 0.070, 0.048, 0.036, 0.029, 0.021 and 0.014 (mM). *V*_max_ value of the competitive inhibition; 0.830 ± 0.011 (nmol/min/mg protein). (b) Secondary plot for calculation of steady-state inhibition constant (*K*_i_) of compound **6h**. *K*_i_ was calculated as 2.07 μM.

**Figure 2.**
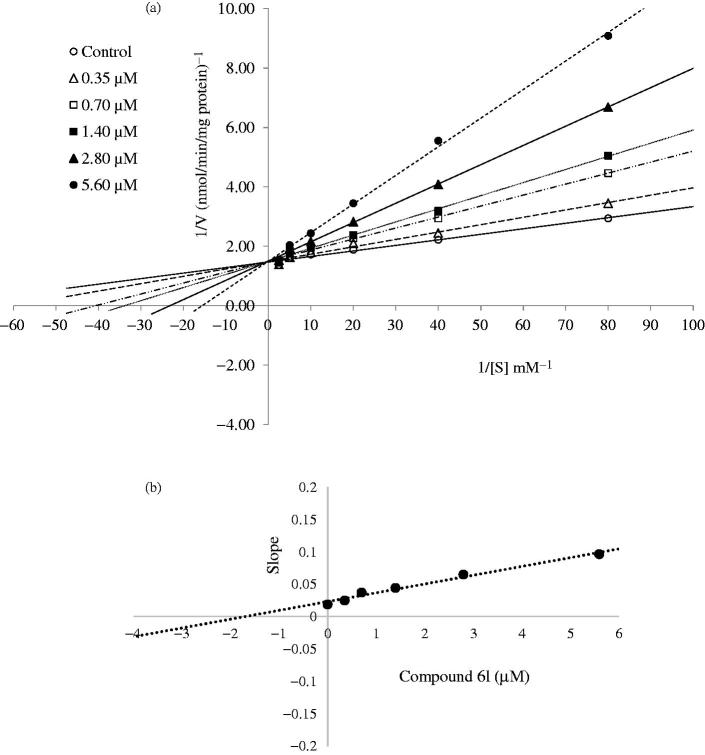
(a) Lineweaver–Burk plots for the inhibition of COX-1 enzyme by compound **6l**. [S], substrate concentration (mM); V, reaction velocity (nmol/min/mg protein). Inhibitor concentrations are shown at the left. *K*_m_ values from 4 × IC_50_ to Control_;_ 0.064, 0.043, 0.030, 0.025, 0.017 and 0.013 (mM). *V*_max_ value of the competitive inhibition; 0.669 ± 0.003 (nmol/min/mg protein). (b) Secondary plot for calculation of steady-state inhibition constant (*K*_i_) of compound **6l**. *K*_i_ was calculated as 1.70 μM.

Substrate dependent kinetic parameters were determined to characterize the mechanism of inhibition of COX isoforms by compounds **6h** and **6l**. The kinetic parameters of this study were determined based on Michaelis–Menten equation followed by a Lineweaver–Burk double reciprocal analysis of data set regarding 1/*V*_max_ versus 1/[S] plot. The Lineweaver–Burk plot analysis of the compounds **6h** and **6l** revealed them as competitive inhibitors. As shown in Figures 1(a) and 2(a), the 1/*V*_max_ (y-intercept) values for five different concentrations (IC_50_/4, IC_50_/2, IC_50_, 2 × IC_50_ and 4 × IC_50_) of compounds **6h** and **6l** are as same as that of no inhibitor, confirming their competitive inhibitory nature for COX-1 on the substrate arachidonic acid. The *K*_i_ (intercept on the x-axis) value of the compounds **6h** and **6l** was determined from the secondary plot of the slope versus varying concentrations (Figures 1(b) and 2(b)). The compounds **6h** and **6l** displayed *K*_i_ values of 2.07 and 1.70 µM for COX-1 enzyme, respectively.

### 3.4. Antimicrobial activity

Synthesized compounds (**6a–6m**) were evaluated for antimicrobial activity against various microorganisms such as *Staphylococcus aureus* (ATCC 25923), *Enterococcus faecalis* (ATCC 29212), *Listeria monocytogenes* (ATCC 1911), *Klebsiella pneumoniae* (NCTC 9633), *Escherichia coli* (ATCC 35218), *E. coli* (ATCC 25922**)***Candida albicans* (ATCC 24433) *Candida krusei* (ATCC 6258) and *Candida parapsilosis* (ATCC 22019). MIC values (Table 2) were revealed by fluorometric measurements using resazurin solution^63^^,^^64^. Chloramphenicol and ketoconazole were used as standard drugs in the activity test. As seen in Table 2, the synthesized compounds (**6a–6m**) have more potency against bacteria than fungi and display similar antibacterial spectrum to the chloramphenicol. The MIC value of 6.25 µg/mL against *E. coli* (ATCC 35218) was observed for all compounds as well as chloramphenicol. Besides, the compounds **6d, 6h, 6l** and **6m** indicated stronger antibacterial activity than the other compounds in the series. These compounds found to be more effective against *Enterococcus faecalis* (ATCC 29212), *Listeria monocytogenes* (ATCC 1911) than chloramphenicol. The compound **6m,** carrying 5-nitrobenzimidazole substructure, was the most active in the series with a better antibacterial spectrum than chloramphenicol. This finding may be explained by the well-known antibacterial effects of 5-nitrobenzimidazoles^65^.

**Table 2.** Antimicrobial activity (MIC μg/mL) of compounds **4**, **6a**–**6m** and reference drugs against pathogenic microorganisms.

Compound	*Sa*	*Ef*	*Lm*	*Kp*	*Ec-1*	*Ec-2*	*Ca*	*Ck*	*Cp*
**4**	50	50	50	50	25	50	100	50	50
**6a**	12.5	12.5	12.5	6.25	6.25	12.5	100	100	100
**6b**	12.5	12.5	12.5	6.25	6.25	12.5	100	100	50
**6c**	12.5	12.5	12.5	12.5	6.25	12.5	100	50	50
**6d**	12.5	6.25	12.5	6.25	6.25	12.5	50	50	50
**6e**	12.5	12.5	12.5	6.25	6.25	12.5	50	50	50
**6f**	12.5	12.5	12.5	12.5	6.25	12.5	100	100	50
**6g**	12.5	12.5	12.5	12.5	6.25	12.5	100	100	50
**6h**	12.5	12.5	6.25	6.25	6.25	12.5	50	100	50
**6i**	12.5	12.5	12.5	6.25	6.25	12.5	100	100	50
**6j**	12.5	12.5	12.5	6.25	6.25	12.5	100	100	50
**6k**	12.5	12.5	12.5	6.25	6.25	12.5	50	50	50
**6l**	12.5	12.5	6.25	6.25	6.25	12.5	50	50	50
**6m**	6.25	6.25	12.5	6.25	6.25	6.25	50	50	50
Chloramphenicol	6.25	6.25	12.5	12.5	6.25	6.25	–	–	–
Ketoconazole	–	–	–	–	–	–	0.78	0.78	0.78

*Sa*: *Staphylococcus aureus* (ATCC 25923); *Ef*: *Enterococcus faecalis* (ATCC 29212); *Lm*: *Listeria monocytogenes* (ATCC 1911); *Kp*: *Klebsiella pneumoniae* (NCTC 9633); *Ec-1*: *Escherichia coli* (ATCC 35218); *Ec-2*: *Escherichia coli* (ATCC 25922); *Ca*: *Candida albicans* (ATCC 24433); *Ck*: *Candida krusei* (ATCC 6258); *Cp*: *Candida parapsilosis* (ATCC 22019)

Antimicrobial activity of the intermediate product 2-(4-methylphenyl)propionic acid (**4**) was also investigated to compare its activity to those of final compounds (**6a–6m**). As seen in the Table 2, the compound **4** is as not active against any bacterial strains.

### 3.5. Cytotoxicity

There are a number of requirements to be fulfilled for successful new drug development. The drug candidate should not only possess intrinsic activity, but should also be able to reach its target and not exhibit toxic effects. Thus, cytoxicity of compounds **6 h** and **6 l**, which demonstrated significant COX inhibition and promising antibacterial activity, was investigated by MTT assay. This assay is based upon the reduction of yellow MTT dye by metabolically active eukaryotic and prokaryotic cells to form the purple formazan product. The assay is generally used to examine cell viability and to estimate cell culture growth^66^^,^^67^. MTT assay was carried out using healthy NIH/3T3 mouse embryonic fibroblast cell lines (ATCC CRL1658), which is recommended for cytotoxicity screening by ISO (10993-5, 2009)^68^. Ibuprofen and nimesulide were also subjected to MTT assay in order to compare cytotoxicity of the compounds **6h** and **6l** with those of reference agents. Table 3 presents the results, in which the synthesized compounds and reference agents displayed IC_50_ of ≥1000 µM. These findings show that the antibacterial activity of the compounds **6h** and **6l** is not due to general toxicity, but can be ascribed to its selective action against bacteria. Furthermore, it may be concluded that the compounds **6 h** and **6 l** are not cytotoxic, because their IC_50_ values against COX enzymes are about 500 fold lower than IC_50_ values against NIH/3T3 cells.

**Table 3.** IC_50_ (μM) values of the ibuprofen. nimesulide and the compounds **6h** and **6l** against NIH/3T3 cell line.

Compound	6h	6l	Ibuprofen	Nimesulide
IC_50_**(**μM**)**	≥1000	≥1000	≥1000	≥1000

### 3.6 Genotoxicity

Ames assay was performed to investigate the genotoxicity of compounds **6h** and **6l**. In Ames^MPF^ assay, more than 25 positive wells were observed with positive controls and negative control wells also showed less than eight positive wells in the presence and absence of S9 with TA98 and TA100, which complied with the requirements for the validation of the Ames^MPF^ and also as described in previous studies^56^. Results are presented in Table 4.

**Table 4.** The AMES^MPF^ results of the compounds.

		Revertants fold increase (over baseline)				
		TA 98		TA 100		
Compound	Concentration (mg/mL)	S9+	S9−	S9+		S9−
**6h**	0.156	0.87	0.56	0.60		0.87
	0.3125	0.52	0.56	0.50		0.52
	0.625	0.70	0.60	0.20		0.70
	1.25	0.17	0.73	0.25		0.17
	2.5	1.05	0.22^a^	0.25		1.05
	5	0.87	0.17^a^	0.20		0.87
**6l**	0.156	0.17^a^	0.57	0.74		0.53a
	0.3125	0.50	0.31^a^	0.52		0.27^a^
	0.625	0.75	0.61	0.52		0.13^a^
	1.25	0.08^a^	0.04a	0.00^a^		0.13^a^
	2.5	0.00^a^	0.00^a^	0.00^a^		0.20^a^
	5	0.00^a^	0.04^a^	0.00^a^		0.00^a^

a *t* Test *p* values (unpaired 1-sided) <.05.

**Figure 3.**
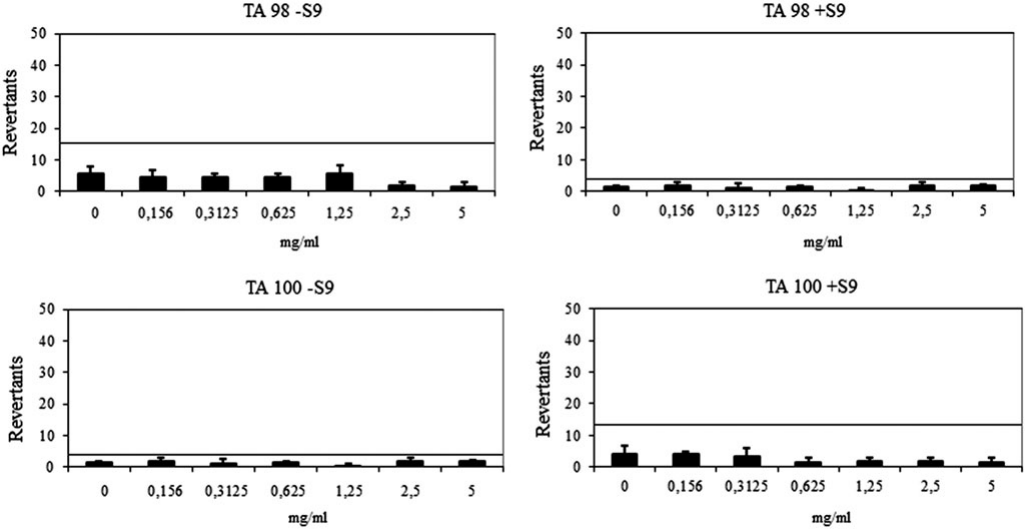
Dose-response curve of compound **6h** against TA98 and TA100 in the presence and absence of S9 according to AMES^MPF^ test.

The compound **6h** showed a baseline of 7.71 with TA98 in the absence of S9 and 1.91 in the presence of S9. Any of the concentrations did not reach the mentioned values above the base-line and also did not show any significance. Therefore, the compound **6h** was classified as non-mutagenic against TA98 in the presence/absence of metabolic activation (S9) (Figure 3). The compound **6h** had a baseline of 1.91 with TA 100 in the absence of S9 and a baseline of 6.65 in the presence of S9. Fold inductions over baseline did not reach values more than 2 or 1.5 and statistically different results did not reveal a dose-response tendency. According to these findings, the compound **6h** did not show any mutagenicity against TA 100 (Figure 3).

**Figure 4.**
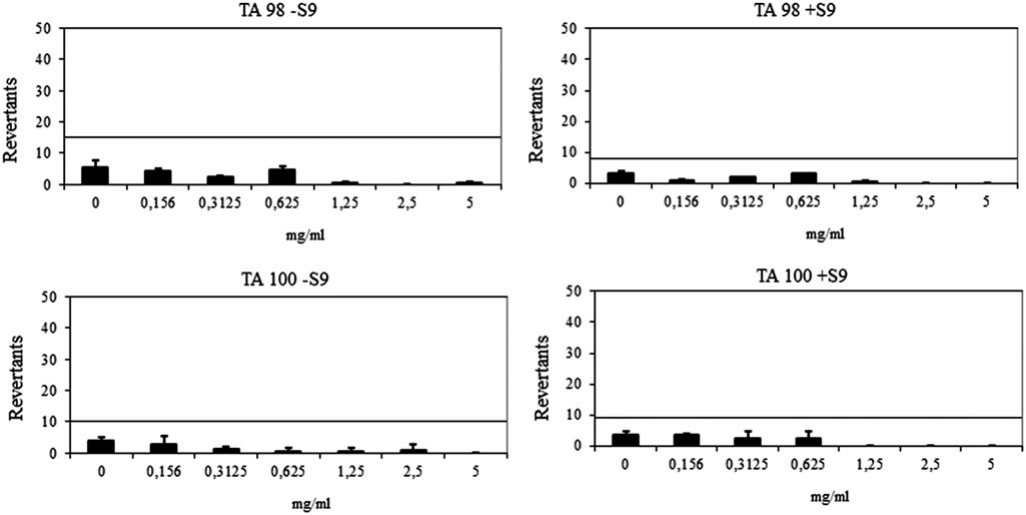
Dose–response curve of compound **6l** against TA98 and TA100 in the presence and absence of S9 according to AMES^MPF^ test.

The compound **6l** showed a baseline of 7.64 and 4.00 against TA 98 with/without S9, respectively. Mentioned-fold increases over the baseline according to the criteria were not determined with the compound **6l**, and significant results did not reach these values and did not show any dose-response tendency. The compound **6l** was also found to be non-mutagenic against TA100 in the presence or absence of metabolic activation (Figure 4). The compound **6l** had a baseline of 5 with TA 100 in the absence of S9 and a baseline of 4.49 in the presence of S9. Fold inductions over baseline were less than 1.5 in each concentration of the compounds and there were not any significant differences. The compound **6l** was accepted as non-mutagenic against TA98 and TA100 with and without metabolic activation (Figure 4). According to the Ames^MPF^ results, the compounds **6h** and **6l** were classified as non-mutagens, which increases the pharmacological importance of the compounds.

### 3.7. Prediction of ADME properties

In addition to essential biological activity, drug candidates should also have an ideal pharmacokinetic profile. Lipinski’s rule evaluates the absorption, distribution, metabolism and elimination (ADME) properties of drug like compounds and is important for the optimization of a biologically active compound. The rule requires that an orally active drug should not have more than one violation^69^. In order to determine pharmacokinetic properties of the synthesized compounds **6a–6m**, the theoretical calculations of the physicochemical parameters (molecular weight (MW), log octanol/water partition coefficient (log P), topological polar surface area (tPSA), number of hydrogen donors (nON), number of hydrogen acceptors (nOHNH), number of rotatable bonds (nRotb) and molecular volume (MV)) are presented in Table 5 along with violations of Lipinski’s rule. According to this data, all of the compounds (**6a–6m**) follow Lipinski’s rule by causing no more than one violation. For compounds **6h** and **6l**, all calculated physicochemical parameters are compatible with Lipinski’s rule. Thus, it may be suggested that synthesized compounds may have a good pharmacokinetic profile, which is crucial for a drug candidate.

**Table 5.** *In silico* physicochemical parameters of the compounds **6a–6m**.

Comp	Log P	tPSA	MW	n ON	nOHNH	nrotb	MV	Vio
**6a**	2.95	49.66	281.40	3	1	5	243.51	0
**6b**	2.73	55.12	276.36	4	1	5	248.54	0
**6c**	2.13	78.87	263.32	5	2	5	227.44	0
**6d**	2.20	68.02	277.35	5	1	5	244.39	0
**6e**	2.06	80.91	278.34	6	1	5	240.23	0
**6f**	4.91	50.19	329.45	3	1	5	281.32	0
**6g**	5.56	50.19	363.89	3	1	5	294.86	1
**6h**	4.94	59.42	359.47	4	1	6	306.87	0
**6i**	4.17	65.98	312.39	4	2	5	275.59	0
**6j**	4.59	65.98	326.42	4	2	5	292.15	0
**6k**	4.82	65.98	346.84	4	2	5	289.13	0
**6l**	4.20	75.22	342.42	5	2	6	301.14	0
**6m**	4.10	111.81	357.39	7	2	6	298.93	0
Ibuprofen	3.46	37.30	206.28	2	1	4	211.19	0
Nimesulide	2.81	101.23	308.31	7	1	5	248.17	0

log P: log octanol/water partition coefficient; tPSA: total polar surface area; MW: molecular weight; nON: number hydrogen acceptors; nOHNH: number of hydrogen donors; nrotb: number of rotatable bonds; MV: moleculer volume; Vio: violation were predicted using molinspiration calculation of molecular properties toolkit.

### 3.8. Molecular docking

**Figure 5.**
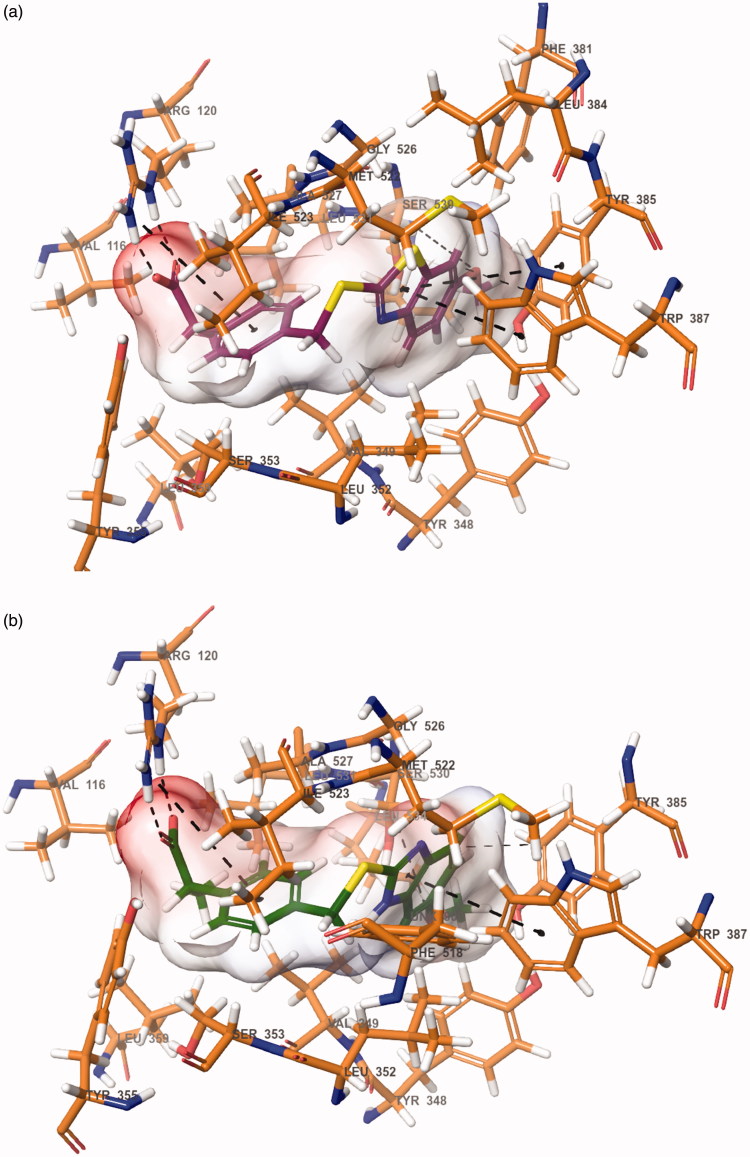
The binding site of COX-1 containing compound **6h** (a) and **6l** (b). The interacting side chain amino acid residues are shown in sticks style.

Docking studies were performed in order to gain more insight into the binding mode of the compounds **6h** and **6l**, and to evaluate the effects of structural modifications on the inhibitory activity against COX-1 enzyme. Studies were carried out by using the X-ray crystal structure of COX-1 enzyme (PDB ID: 1EQG)^10^ obtained from Protein Data Bank server (www.pdb.org). The docking poses of the compounds **6h** and **6l** are presented in Figure 5(a,b).

When the docking studies are analyzed, it is seen that the inhibitor ibuprofen binds in the COX active site, which is consisted of a long narrow hydrophobic channel lining from the membrane binding surface to the center of the protein. The propionic acid group of ibuprofen is very essential in terms of binding to the active site. This group takes part in a network of polar interactions, which include two hydrogen bonds between the propionic acid (carbonyl and hydroxyl groups) and Arg120^8^^,^^10^.

The compounds **6h** and **6l** are settled in the hydrophobic channel very concordantly, likewise ibuprofen. Phenyl propionic acid is the common group of the ibuprofen and the compounds **6h** and **6l**. Propionic acid moiety forms two hydrogen bonds with Arg120. Furthermore, the phenyl ring constitutes a salt bridge with Arg120. Benzothiazole and benzimidazole provide aromaticity for compounds **6h** and **6l**, respectively. These structures interact with the phenyl of Tyr385 and indole of Trp387 by doing π–π interactions in both compounds **6h** and **6l**.

In terms of chemical structures of the synthesized compounds (**6a**–**6m**), only the compounds **6h** and **6l** have methoxy substituents in fifth position of benzothiazole and benzimidazole. The methoxy group ensures significant polar interaction with the amino group of Leu534 by doing a hydrogen bond. By virtue of this interaction, compounds **6h** and **6l** could bind to the active site, efficiently and may have a higher COX inhibition potency than other derivatives in the series.

### 3.9. Structure activity relationships (SARs)

The substitution pattern was explored using various (benz)azolylthio moieties in 2-[4-methylphenyl]propionic acid main substructure. Thus, determination of contribution of the various bioisosteric (benz)azolylthio moieties to COX inhibitory and/or antimicrobial activity and evaluation of SARs were planned. The noteworthy results of enzyme inhibition, antimicrobial, physicochemical parameters calculation and docking studies also required to discuss structure activity relationships (SARs). However, SARs cannot be discussed for antifungal activity due to high MIC values of the compounds (**6a**–**6m**). Moreover, observation of very similar antibacterial activity, displayed by the compounds (**6a**–**6m**) indicates that there is no important difference between contributions of azolylthio moieties to antibacterial activity and makes consideration of the SARs very difficult. Only presence of the 5-nitro substitution benzimidazolylthio moiety in compound **6l** results with enhanced antibacterial activity. Hence, it can be assumed that promising antibacterial activity of the compounds (**6a**–**6m**) is related to their general structural characteristics. Lower antibacterial activity results, observed in the compound **4**, also support this approach and highlight the importance of (benz)azolylthio moiety on antibacterial activity.

Against COX enzymes, all target compounds (**6a**–**6m**) exhibited better COX inhibition than intermediate compound **4**. This finding displays that incorporation of (benz)azolylthio and 2-(4-methylphenyl)propionic acid structures has a positive contribution to COX inhibitory activity. However, the compounds **6a**–**6e** have lower inhibition potency than the compounds **6f**–**6m**. The first suggestion of this observation can be the logP values of the compounds. Increasing logP in compounds **6f**–**6m** may enhance the enzyme inhibition potency (Table 5). Second, it can be suggested that in the compounds **6f**–**6m** presence of a benzazolylthio moiety, which is absent in **6a**–**6e**, promotes the enzyme inhibition as a result of π–π interaction in the active site of enzyme. Among the compounds **6f**–**6m**, the most active compounds are **6 h** and **6 l.** The common feature of the **6h** and **6l** separating from other compounds is a methoxy substituent in the fifth position of benzothiazolylthio and benzimidazolylthio substructures. Thus it may be suggested that methoxy group creates more inhibition potency than the other substituents. This proposal may be explained by hydrogen accepting ability of alkyloxy groups and has been supported by the docking study (Figure 5(a,b)). It is well known that 2-phenylpropionic acid is the main substructure, being responsible to COX inhibition, in lots of well-known marketing drugs. Thus, this substructure has been fixed in all compounds. Importance of 2-phenylpropionic acid in COX inhibition has also been observed in the docking studies (Figure 5(a,b)).

## 4. Conclusions

In summary, preliminary evaluation of new 2-(4-substitutedmethylphenyl)propionic acid derivatives as dual COX inhibitory-antibacterial agents resulted with promising findings. The compounds **6h** and **6l** displayed a good antibacterial profile along with significant COX-1 and COX-2 inhibition. Furthermore, these compounds did not show cytotoxicity and mutagenicity. Docking study indicated the significant interactions between both compound**s** and COX-1 enzyme. Consequently, findings of this study will not only direct our research group to further studies, but also may have an impact on medicinal chemists, stimulating them to synthesize more effective and safer compounds bearing chemical structures similar to those of the compounds **6h** and **6l** as dual COX inhibitory-antibacterial agents.
